# Definitive Interstitial Brachytherapy for Isolated Cutaneous Metastasis in Breast Cancer

**DOI:** 10.7759/cureus.114383

**Published:** 2026-08-11

**Authors:** Chandra Prakash Verma, Pamela Sen, Abhilash Banerjee

**Affiliations:** 1 Department of Radiation Oncology, Institute of Medical Sciences, Banaras Hindu University, Varanasi, IND; 2 Department of Radiation Oncology, Yashoda Hospital, Hyderabad, IND

**Keywords:** breast cancer, high dose rate brachytherapy, interstitial brachytherapy, isolated cutaneous metastasis, oligometastatic disease

## Abstract

Cutaneous metastasis as the sole site of recurrence in breast cancer is rare and poses a unique therapeutic challenge due to limited consensus on optimal management strategies. We present the case of a 40-year-old female with isolated cutaneous metastasis over the left side of the neck from previously treated breast cancer, presenting 1.5 years after completion of primary therapy. Following evaluation, the patient was treated with interstitial high-dose-rate brachytherapy, followed by maintenance systemic therapy with anastrozole and palbociclib. At six months of follow-up, she achieved a complete clinical response with minimal toxicity. This case highlights the potential role of interstitial brachytherapy as an effective, organ-preserving treatment modality in selected patients with isolated cutaneous metastasis.

## Introduction

Cutaneous metastasis is an uncommon manifestation, occurring in approximately 0.7-10.4% of patients with metastatic cancer and accounting for nearly 2% of all cutaneous tumors [1]. The most common malignancy to metastasize to the skin is malignant melanoma, followed by breast cancer.

Breast cancer accounts for up to 23.9% of all cutaneous metastases [2], typically presenting within the first three years of diagnosis and most commonly affecting patients between 50 and 70 years of age [1]. However, isolated distant cutaneous metastasis is rare, particularly in the absence of systemic disease. Furthermore, solitary involvement of the head and neck region is exceedingly uncommon.

Management of isolated cutaneous metastasis is not standardized, with options including surgical excision, electron beam radiotherapy, and interstitial brachytherapy. Interstitial brachytherapy allows highly conformal, tissue-sparing dose delivery, making it particularly useful for lesions close to critical structures. Herein, we report a rare case of isolated cutaneous metastasis over the neck in a patient with previously treated breast cancer, successfully managed with interstitial brachytherapy.

## Case presentation

We present the case of a 40-year-old premenopausal female with a history of infiltrating ductal carcinoma of the left breast, hormone receptor-positive and human epidermal growth factor receptor 2 (HER2)-negative, diagnosed four years prior. She underwent modified radical mastectomy (MRM), with histopathology showing moderately differentiated invasive ductal carcinoma, not otherwise specified (NOS), with free margins, lymphovascular invasion, and perineural emboli. Axillary dissection revealed 11 of 17 lymph nodes positive with extranodal extension (ENE), pathologically staged as pT2N3a (Stage IIIC). Given the high nodal burden and ENE positivity, she received adjuvant chemotherapy with six cycles of three-weekly TAC (docetaxel 75 mg/m², doxorubicin 50 mg/m², cyclophosphamide 500 mg/m², all on day 1), followed by adjuvant conventional radiation via Cobalt-60 teletherapy to the left chest wall, supraclavicular fossa, and axilla (40 Gy/15 fractions over three weeks), and was subsequently maintained on hormonal therapy.

**Figure 1:**
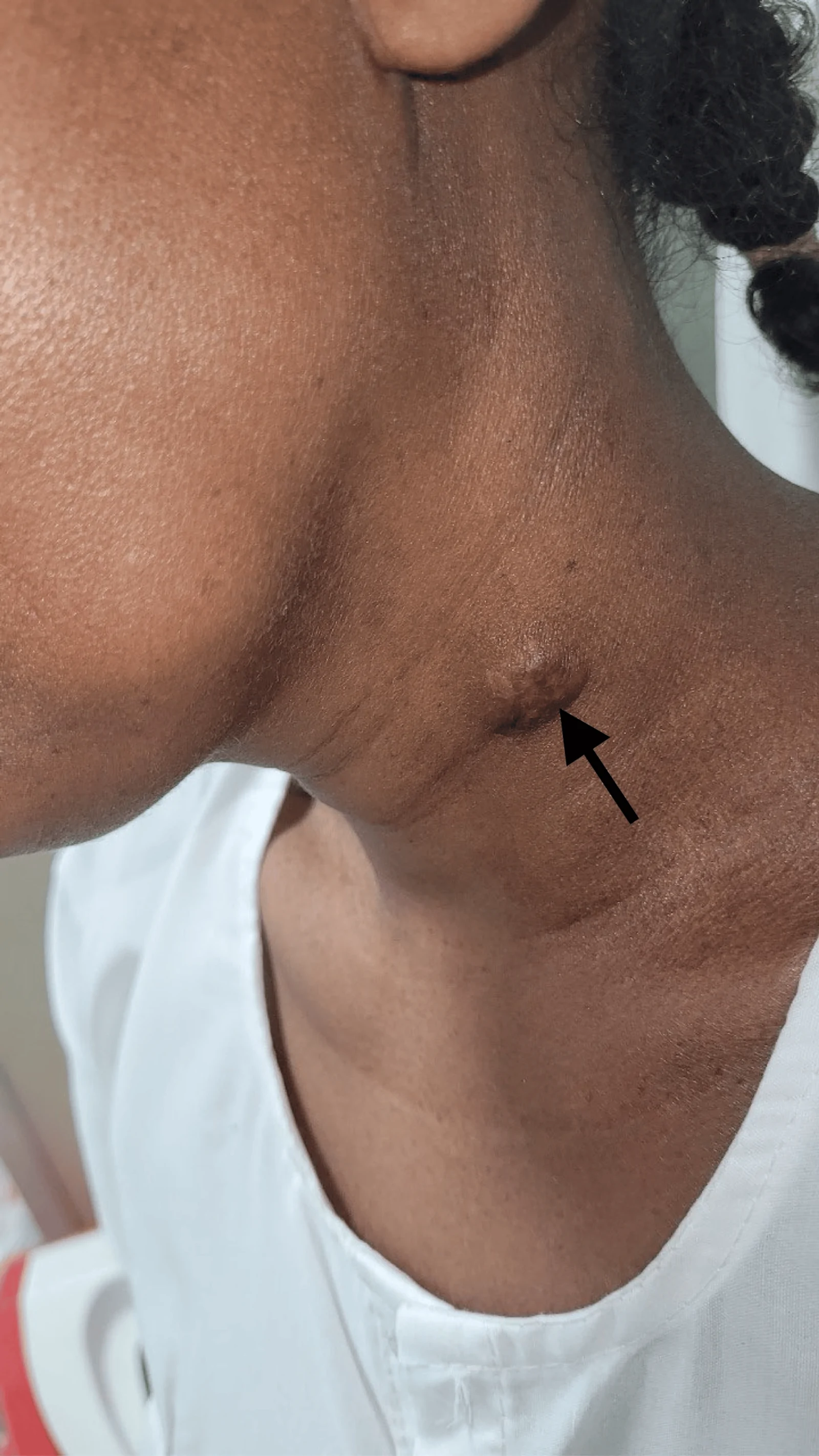
Well-defined skin-coloured nodule over the left side of the neck

She remained on regular follow-up for 1.5 years before presenting with a firm cutaneous nodule over the neck. Clinical examination revealed a single, well-defined, asymptomatic, skin-coloured to erythematous nodule over the left side of the neck, measuring approximately 1.5 × 1.5 cm (Figure 1), located outside the previously irradiated field.

**Figure 2:**
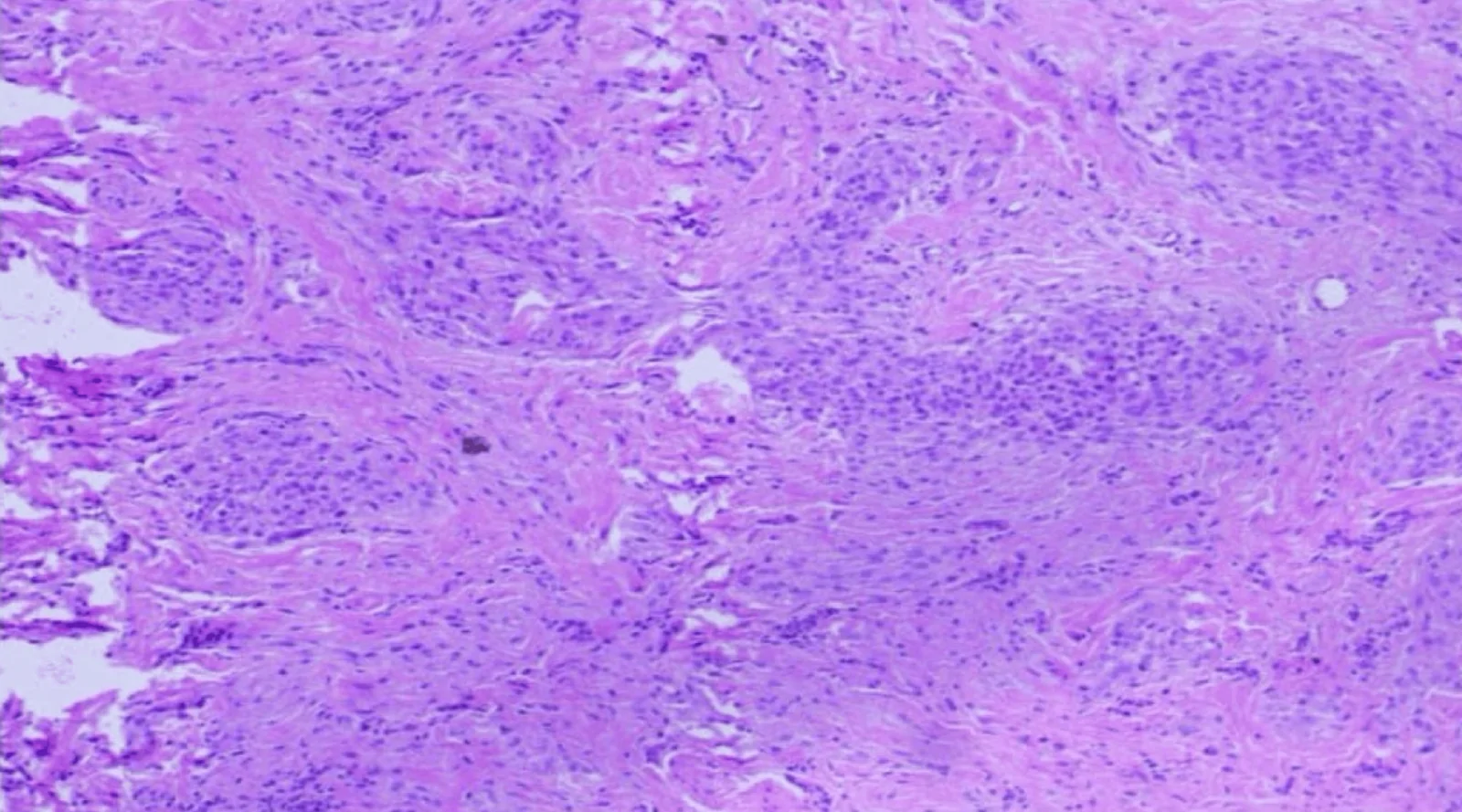
Histopathological examination of incisional skin biopsy showing metastatic deposit likely from breast primary

Incisional biopsy confirmed metastatic carcinoma consistent with a breast primary (Figure 2).

Metastatic workup, including chest X-ray and ultrasound of the whole abdomen, showed no evidence of disease. Ultrasound of the neck confirmed a solitary 1.5 × 1.5 × 0.8 cm cutaneous nodule on the left side of the neck, with no additional lesions or pathological cervical lymphadenopathy. These findings supported a diagnosis of isolated cutaneous metastasis from breast cancer. After multidisciplinary tumor board discussion, definitive interstitial brachytherapy was planned, given its sharp dose fall-off and ability to limit toxicity to underlying structures. Surgery was reserved for residual or recurrent disease, if required.

**Figure 3:**
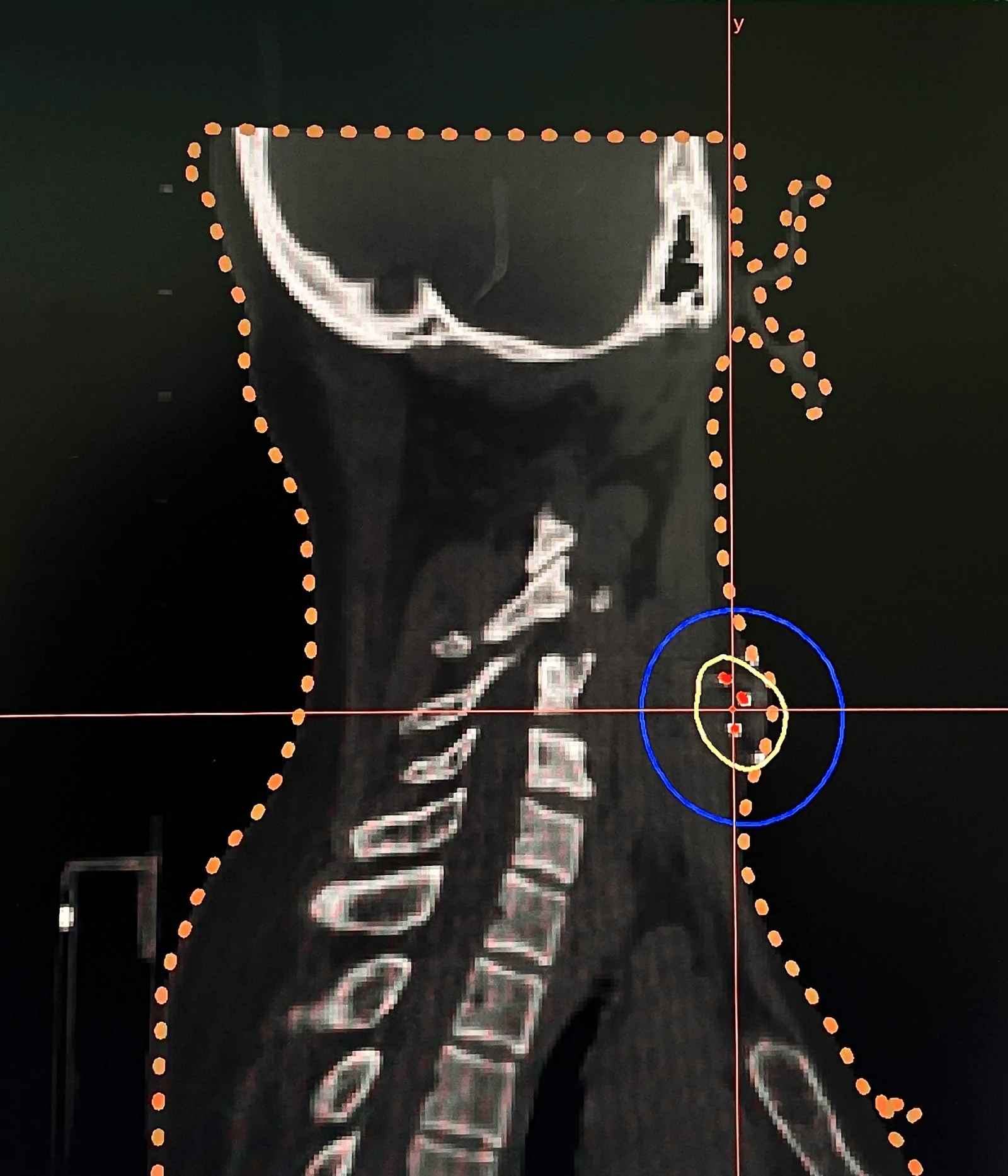
CT-based treatment plan showing catheter placement within the target lesion, with the 100% isodose line (yellow) closely conforming to the clinical target volume and the 30% isodose line (blue) demonstrating the steep dose fall-off sparing surrounding tissue.

The patient was positioned supine with the head turned to the right. Under ultrasound guidance, three stainless steel needles were inserted into the lesion, spaced 7.5 mm apart and aligned parallel to each other. Plastic catheters were then inserted and secured to the skin using buttons. The clinical target volume (CTV) was delineated on planning CT images, and adequate coverage by the 100% isodose line was ensured (Figure 3).

A total dose of 36 Gy in six fractions was delivered, with two fractions per week over three weeks, using an Iridium-192 microSelectron® high-dose-rate brachytherapy system (Elekta, Stockholm, Sweden).

**Figure 4:**
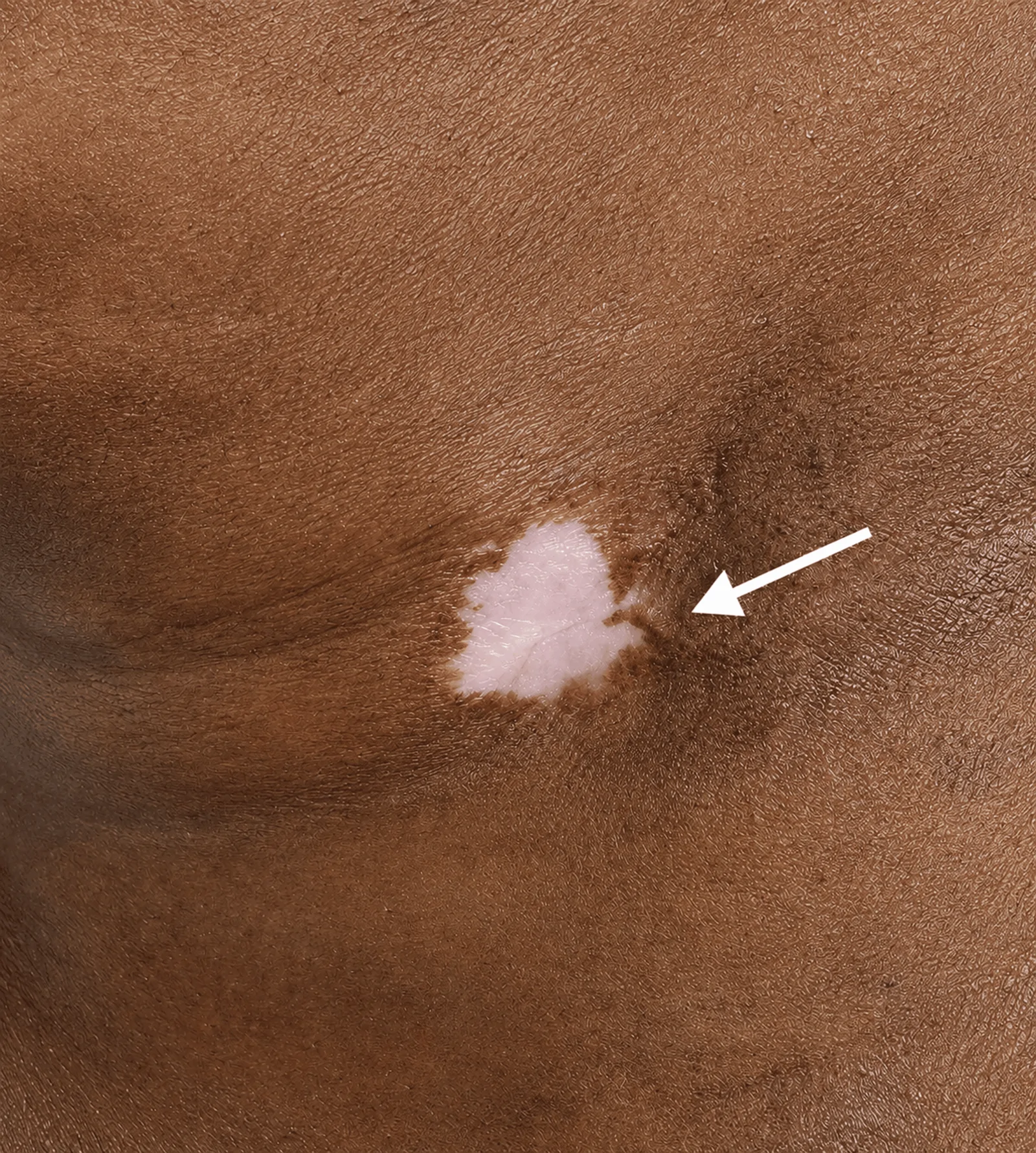
Small hypo-pigmented patch 6 months after brachytherapy

Skin toxicity was monitored throughout treatment. The patient developed a grade II skin reaction in the last week of brachytherapy, managed conservatively with topical emollients and skin care measures, which resolved by four weeks post-treatment. One month after completion of brachytherapy, she was initiated on systemic therapy with anastrozole and palbociclib, planned to continue until disease progression or intolerance. At six months post-treatment, she achieved a complete clinical response, with only a small residual hypopigmented patch (Figure 4).

She continues to remain disease-free at 2 years of follow-up.

## Discussion

Metastasis refers to a neoplastic lesion that has spread from another tumour with which it is no longer contiguous or is not in close proximity within the same tissue plane. Cutaneous metastasis accounts for 0.7-9% of all metastatic disease and may represent the earliest sign of recurrence in a previously treated malignancy, or may unmask an occult internal primary [1]. Spread to the skin can occur through lymphatic or haematogenous routes, by direct extension, or, uncommonly, through iatrogenic implantation [3]. Breast cancer is responsible for up to 23.9% of all cutaneous metastases, yet only a small proportion of breast cancer patients ever develop skin involvement, and in rare instances, as in the present case, it is the sole indicator of relapse. The anterior chest wall is the most frequently affected site, followed by the abdominal wall, while presentation in the head and neck remains distinctly uncommon [4]. Hormone receptor-positive tumours tend to metastasize to the skin in isolation, present after a longer latency period, follow a more indolent course, and are associated with better survival than other molecular subtypes [5].

A comparable presentation was reported by Fava et al. (2023), who described an isolated cutaneous metastasis of the scalp arising 15 years after initial treatment for breast cancer [6]. Together, these two cases demonstrate that cutaneous metastasis can be the only sign of disease recurrence even after a long disease-free interval, and that its resemblance to benign skin lesions can delay diagnosis. Cutaneous metastases are typically classified according to their timing relative to the primary tumour (early versus late), their site (local versus distant), and the clinical context in which they occur (occult breast malignancy versus established recurrence). Although head and neck or scalp involvement accounts for only a small proportion of cases, these sites carry prognostic relevance, as hormone receptor-positive disease at these locations often follows a slower, more favourable course [5].

No established standard of care exists for the management of cutaneous metastasis, and current treatment approaches are largely extrapolated from evidence in oligometastatic non-small-cell lung, prostate, and oesophageal cancers. In these settings, incorporating definitive local therapy alongside maintenance systemic treatment has been shown to improve both overall survival (OS) and progression-free survival (PFS)[7]. In breast cancer patients whose disease is confined to the skin without visceral involvement, metastasis tends to manifest later than in other organs, survival may be considerably prolonged, and cure is achievable in selected cases. A report using high dose rate Ir-192 interstitial brachytherapy in two breast cancer patients with loco-regional recurrence found favourable in-field control of a skin metastasis in one patient, though with persistent local sequelae and subsequent outfield metastases, while another patient with a solitary axillary nodal metastasis was successfully treated [8]. In a retrospective series, CT-guided iodine-125 brachytherapy in patients with local-regional recurrent breast cancer achieved an objective response rate of 66.7% and local control rate of 93.3% at six months, with no severe complications [9].

In our patient, interstitial brachytherapy was selected in preference to electron beam radiotherapy because it allows highly conformal dose delivery directly into or immediately around the target lesion, with a steep dose fall-off that spares surrounding healthy tissue. Electron beam radiotherapy, though non-invasive and technically simpler, offers less precise depth-dose control and a broader penumbra near critical structures, and typically requires a wider treatment field than needed for a small, localized lesion. Interstitial brachytherapy, by contrast, allows the dose distribution to be tailored to irregularly shaped tumours, is less affected by patient motion, permits dose escalation, and reduces exposure of nearby organs at risk. For cutaneous lesions of the neck in particular, these features are valuable for limiting toxicity to underlying structures such as the thyroid gland and cervical musculature, with a favourable cosmetic outcome achieved in this case. Given these advantages, together with the ability to achieve high local control within a short treatment course, interstitial brachytherapy was chosen as the preferred treatment modality in our patient.

## Conclusions

Cutaneous lesions can be the first sign of tumour metastasis, and prompt recognition allows successful salvage. In our patient, interstitial high-dose-rate brachytherapy with maintenance anastrozole and palbociclib achieved a complete clinical response by six months, with a good cosmetic outcome and no significant toxicity. High-dose-rate brachytherapy, therefore, appears to be a promising approach in carefully selected patients with isolated cutaneous malignant lesions, though further evidence from larger studies is needed to confirm its efficacy and define its role in clinical practice.
